# Transcriptional control of two distinct lactococcal plasmid-encoded conjugation systems

**DOI:** 10.1016/j.crmicr.2024.100224

**Published:** 2024-02-05

**Authors:** Guillermo Ortiz Charneco, Philip Kelleher, Andrius Buivydas, Paul P. de Waal, Irma M.H. van Rijswijck, Noël N.M.E. van Peij, Jennifer Mahony, Douwe Van Sinderen

**Affiliations:** a School of Microbiology & APC Microbiome Ireland, University College Cork, Western Road, Cork, Ireland; b dsm-firmenich; Taste, Texture & Health, Center for Food Innovation, Alexander Fleminglaan 1, 2613 AX Delft, the Netherlands

**Keywords:** Lactococci, Conjugative plasmid, Promoter, Primer extension, Transcription, Regulation, EMSA

## Abstract

Lactococcal conjugative plasmids are poorly characterized compared to those harbored by numerous other Gram-positive bacteria, despite their significance in dairy fermentations and starter culture development. Furthermore, the transcriptional landscape of these lactococcal conjugation systems and their regulation have not been studied in any detail. Lactococcal plasmids pNP40 and pUC11B possess two genetically distinct and prevalent conjugation systems. Here, we describe the detailed transcriptional analysis of the pNP40 and pUC11B conjugation-associated gene clusters, revealing three and five promoters, respectively, for which the corresponding transcriptional start sites were identified. Regulation of several of these promoters, and therefore conjugation, is shown to involve the individual or concerted activities of the corresponding relaxase and transcriptional repressor(s) encoded by each conjugative plasmid. This work highlights how the conjugative potential of these systems may be unlocked, with significant implications for the starter culture and food fermentation industry.

## Highlights

• Active promoters in pNP40 and pUC11B conjugation clusters are identified.
• Regulatory elements of said clusters include the conjugation-associated relaxases.
• Relaxase expression of both systems appears to be autoregulated.
• Tra20 may regulate pNP40 conjugation in an uncharacterized manner.

## Graphical abstract

**Figure:**
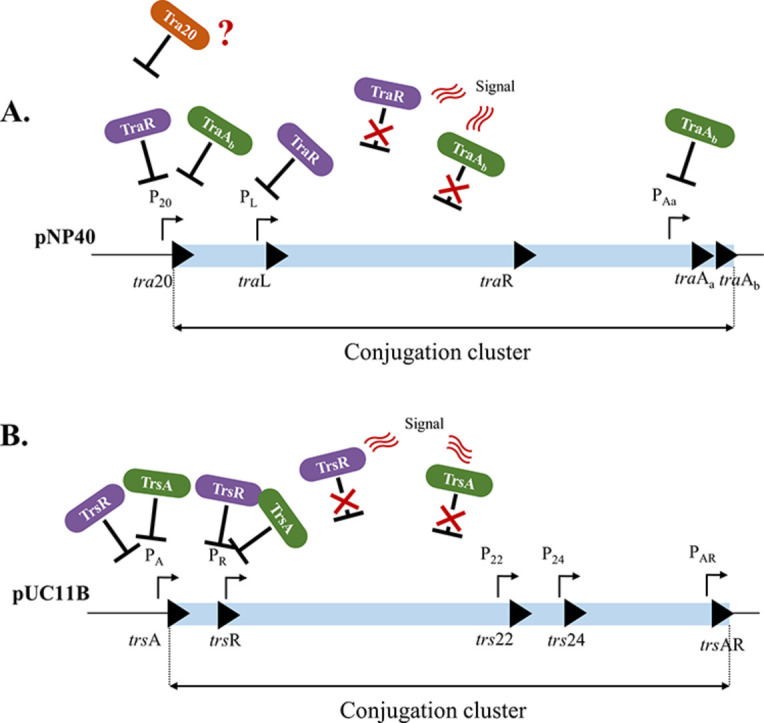

## Introduction

Plasmids are extra-chromosomal DNA elements, which are widespread among lactic acid bacteria (LAB), and which are known to harbor genes that confer several industrially relevant traits, such as lactose metabolism (Mills et al., 2006), proteolysis, bacteriophage resistance (Ainsworth et al., 2014), bacteriocin and exopolysaccharide production, and conjugative transfer (Fallico et al., 2012). Conjugation among Gram-positive bacteria is a biological phenomenon involving the transfer of (in most cases) single-stranded DNA (ssDNA) from a donor to a recipient cell, via a molecular machinery encoded by genes that tend to be organized in a single gene cluster (Kohler et al., 2019). The basic conjugation machinery of Gram-positive species differs from that of Gram-negative organisms in that donor-recipient cell-to-cell contact in the former is stablished via surface adhesins instead of conjugative pili (for the latter), while they also lack an outer membrane core complex due to fundamental differences in their cell envelope structure (Grohmann et al., 2018).

The conjugative process is believed to be strictly regulated to minimize the fitness cost associated with the maintenance of the conjugative machinery, while also maximizing the flexible benefits of self-transmission. Regulation of pLS20-mediated conjugation in *Bacillus subtilis* was revealed to be a multi-layered and complex system consisting of an interplay between three distinct proteins that co-act in controlling the activity of the main conjugation promoter, which is tightly repressed in a manner that nonetheless allows fast activation (Meijer et al., 2021). Similarly, the Gram-positive conjugative plasmid pIP501, which was originally isolated from *Streptococcus agalactiae* (Horodniceanu et al., 1976), has served as the model system for Gram-positive conjugation due to its relatively small size and basic organization. The conjugation gene cluster of this plasmid was shown to be organized as two transcriptional units, with the first encoding the main conjugation-associated functions (relaxase, mating channel formation, ATPases, etc.) and the second encoding the surface adhesin and the transcriptional repressor of the system (Kurenbach et al., 2002). In the pIP501 conjugation system, *traO* encodes the surface adhesin and *traN* specifies a repressor protein that regulates its own transcription and that of *traO* by binding to the promoter-containing region of the second transcriptional unit. It also binds to the promoter-encompassing region of the first transcriptional unit, which comprises the *tra* region, thereby negatively regulating transcription of the whole conjugation gene cluster (Kurenbach et al., 2006; Kohler et al., 2019).

Lactococcal plasmid-encoded conjugation gene clusters can be broadly divided in two distinct groups. The first one, the so-called pNP40-like, are the most prevalent group based on public database searches (Ortiz Charneco *et al*., 2021). The second group, referred to as the pMRC01-like, encompasses plasmids such as pMRC01 (Dougherty et al., 1998), pAF22 (Fallico et al., 2012) and pUC11B, each presenting significant amino acid identity to each other, while still presenting notable differences in their genetic contents and gene organization (Ortiz Charneco *et al*., 2021). Despite their sequence disparity, both pNP40 and pUC11B have been determined to possess the essential genes required for conjugation, such as a relaxase, two ATPase-like/associated proteins and at least one mating channel component, as well as a transcriptional repressor that may regulate the expression of these conjugation clusters (Ortiz Charneco *et al*., 2023).

Although the functions of the genetic elements associated with lactococcal conjugation systems have recently been more clearly defined, the transcriptional landscape of these elements and their possible regulation remain unclear. To address this knowledge gap, transcriptional analysis to determine the active promoter regions within each conjugation cluster, as well as the precise transcriptional start sites was investigated and is described herein. Conjugation frequencies of plasmid pNP40 have been reported to be relatively low (in the order of 3–5 × 10^–4^%; Harrington and Hill, 1991, Trotter et al., 2001). More recent conjugation studies using pNP40 and pUC11B (Ortiz Charneco *et al*., 2021, 2023) have not only described relatively high pUC11B-mediated conjugation frequencies and a significant increase of previously reported pNP40-mediated conjugation frequencies, but have also reported that these frequencies varied significantly depending on the applied conditions, for example displaying no discernible conjugation under liquid mating conditions, all suggesting that conjugation is a regulated phenomenon. Moreover, two putative transcriptional repressor-encoding genes were identified in the conjugation gene clusters of pNP40 and pUC11B, represented by *traR* and *trsR*, respectively. Mutations in either of these genes was shown to cause significantly increased conjugation frequencies, indicating that conjugation is governed through negative transcriptional regulation. These findings prompted a transcriptional analysis of these two conjugation gene clusters.

## Materials and methods

### Bacterial strains, plasmids and growth conditions

Experiments were predominantly conducted using derivatives of *Lactococcus cremoris* NZ9000 (Kuipers et al., 1998) harboring (derivatives of) either plasmid pNP40 or pUC11B, which had been generated by conjugation (and subsequent recombineering) in previous studies (Ortiz Charneco *et al*., 2021, 2023). A complete list of bacterial strains used in this study can be found in Supplementary Table S1. Overnight bacterial cultures were grown at 30 ºC for 16 h and were prepared by inoculating cells into 10 mL of M17 + 0.5% (v/v) glucose (GM17) containing either nisin (2.5 μg/mL, for selection of pNP40), tetracycline (10 μg/mL, to select strains harboring pUC11B) and/or erythromycin (5 μg/mL, to select for strains containing erythromycin resistance-conferring derivatives of pPTPL and pNZ8048, i.e. pPEPL and pNZ8048E). Electrocompetent cells of L. *cremoris* were prepared as previously described (Holo and Nes, 1989).

Salient features of plasmids and their derived constructs created in this study are summarised in Supplementary Table S2. Plasmid constructs were generated via conventional recombinant DNA techniques. PCR fragments used for cloning were digested with the same enzymes as the relevant vector and ligated with T4 DNA ligase (New England Biolabs, Ipswich, MA, USA). For the construction of plasmid pNZ8048E, the backbone of pNZ8048 (De Ruyter *et al*., 1996) without the chloramphenicol resistance gene and the erythromycin resistance gene from pNZ44E (Draper et al., 2009) were PCR-amplified using the oligonucleotides listed in Supplementary Table S3 (pNZ8048-Fw/Rv and Ery-Fw/Rv) and ligated into the EcoRI and BsrGI recognition sites. Similarly, for the construction of plasmid pPEPL, the backbone of pPTPL (O'Driscoll *et al*., 2004), excluding the *tetK* gene (pPTPL-Fw/Rv), and the erythromycin resistance gene from pNZ44E were PCR amplified and restricted with BsrGI and XhoI prior to ligation. The integrity of all newly generated constructs was verified by Sanger sequencing (Eurofins, Ebersberg, Germany).

### Protein overexpression and purification

Genes encoding Tra20, TraR, TraA_a_ and TraA_b_ from pNP40, as well as those encoding TrsA and TrsR from pUC11B, were cloned under the control of the nisin-inducible promoter of the high copy number pNZ8048E expression vector, using the oligonucleotides listed in Supplementary Table S3. Oligonucleotides were designed to incorporate either a C-terminal (TraA_a_, TraA_b_ and TrsA) or an N-terminal (Tra20, TraR and TrsR) hexa-histidine-encoding tag in each protein-encoding sequence. These plasmid constructs were introduced in L. *cremoris* NZ9000 by electroporation.

L. *cremoris* NZ9000 cells harboring the different pNZ8048E-derived plasmid constructs were incubated overnight at 30 ºC in GM17 broth supplemented with 5 μg/mL erythromycin. Thereafter, 800 mL GM17 similarly supplemented with erythromycin was inoculated with a 1.5% inoculum of the different cultures and incubated at 30 ºC until an OD_600_ ≈ 0.2 was achieved. Expression of the recombinant proteins was induced by adding 10 ng/mL nisin, followed by continued incubation at 30 ºC for 3.5 h. Cells were then harvested by centrifugation (8000 × *g*, 15 min, 4 °C), washed and concentrated 40-fold in wash buffer (10 mM Tris–HCl, 50 mM CaCl_2_, 300 mM NaCl, 20 mM imidazole; pH 7.5). To lyse the cells, the cell pellets were resuspended in 20 mL lysis buffer (10 mM Tris–HCl, 50 mM CaCl_2_, 300 mM NaCl, 10 mM imidazole, 30 mg/mL lysozyme) and incubated at 37 ºC for 30 min. The lysis mixture was then sonicated five times in 30 second bursts. Sonicated cells were centrifuged at 17,000 × *g* for 25 min at 4 ºC to remove cell debris. Protein purification was achieved using His-tag affinity gravity-flow chromatography, using Ni-NTA matrices in accordance with the manufacturer's instructions (Qiagen), and the imidazole present in the final elution was removed using a dialysis membrane with a molecular weight cut-off of 12–14 kDa, diameter 19 mm (Medicell Membranes Ltd, London, UK). Final elution fractions were then analysed by SDS polyacrylamide gel electrophoresis as previously described (Laemmli, 1970), on 10–12.5% polyacrylamide gels. Gels were run at 160 V for 90 min. Following electrophoresis, gels were fixed and stained with Coomassie Brilliant Blue, and final protein concentrations were estimated using the Bradford Assay (Kielkopf et al., 2020).

### Electrophoretic mobility shift assay

The ability of a (purified) protein to specifically bind to a DNA fragment encompassing a given promoter region from the conjugation gene cluster of either pNP40 or pUC11B was determined by Electrophoretic Mobility Shift Assay (EMSA). DNA fragments representing different portions of the promoter regions upstream of *tra20, traL, traA_a_, trsA* and *trsR* were prepared by PCR using IRD700-labelled primers pairs (Integrated DNA Technologies, Coralville, IA, USA) (Supplementary Table S3). EMSAs were performed essentially as previously described (Hamoen et al., 1998; O'Connell Motherway *et al*., 2011), with minor modifications as follows. Binding reactions were carried out in a final volume of 25 µL in the presence of poly[d(I-C)] in binding buffer (20 mM Tris–HCl, 10 mM MgCl_2_, 0.5 mM DTT, 1 mM EDTA, 100 mM KCl, 10% glycerol). Varying concentrations of purified Tra20, TraR, TraA_a_, TraA_b_, TrsA and TrsR ranging from 10 nM to 250 nM, and a fixed amount of DNA probe (0.1 pmol) were mixed and immediately incubated for 30 min at 30 ºC. Samples were loaded on 6% non-denaturing Polyacrylamide (PAA) gel prepared in TAE buffer (40 mM Tris acetate [pH 8.0], 2 mM EDTA) and run in a 0.5 x TAE at 100 V for 110 min in an Atto Mini PAGE system (Atto Bioscience and Biotechnology, Tokyo, Japan). Signals were detected using a ChemiDoc MP Imaging System (Bio-Rad) and captured using the supplied Image Lab Software. Details pertaining to each probe used for EMSAs are outlined in Supplementary Table S4.

### Non-selective recombineering

Using a previously described ssDNA recombineering approach (van Pijkeren and Britton, 2012), specific oligonucleotides for *traA_b_* (pNP40) and *trsA* (pUC11B) were designed (Supplementary Table S3) to insert two in-frame stop codons flanking a restriction endonuclease (RE) recognition site upon incorporation. For the purpose of generating double recombinants (plasmids harboring separate mutations in two different genes within their conjugation clusters), the recombinant strains generated in previous studies, i.e. L. *cremoris* NZ9000 pJP005, pNP40 with either *tra20, traR, traA_a_* or *traA_b_* mutated (nomenclature *tra*_pNP40_::Ter) and L. *cremoris* NZ9000 pJP005, pUC11B carrying a mutation in either *trsA* or *trsR* (nomenclature *trs*_pUC11B_::Ter) were used (Ortiz Charneco *et al*., 2021, 2023). For recombineering purposes, 400 μg of recombineering oligonucleotide and 45 μL of electrocompetent cells harboring the previously mutated versions of the conjugative plasmids, *trsR*_pUC11B_::Ter or *traR*_pNP40_::Ter and pJP005, were electroporated at 2000 V, 25 μF, and 200 Ω. Bacterial cells subsequently recovered for 2.5 h at 30 ºC, after which they were serially diluted and plated on GM17 agar plates containing 10 μg/mL tetracycline for pUC11B selection (double mutant nomenclature *trsA/trsR*_pUC11B_) or 2.5 μg/mL nisin for pNP40 selection (double mutant designated as *traR/traA_b-_*_pNP40_).

After 48 h of incubation, recombinant colonies were screened by mismatch amplification mutation analysis-PCR (MAMA-PCR) of individual randomly selected colonies (Cha et al., 1992; van Pijkeren and Britton, 2012). Once a positive recombinant genotype was identified, the relevant colony was streaked on GM17 agar plates supplemented with tetracycline or nisin to isolate a derivative with a pure genotype. Using this pure culture, a 1 kb fragment spanning 500 bp upstream and downstream of the recombinant sequence was amplified by PCR and the incorporation of the mutation validated by Sanger sequencing (Eurofins, Ebersberg, Germany).

### Mapping of transcription start sites by *lacZ* gene fusions and β-galactosidase assays

To determine the presence of promoters within the pNP40 and pUC11B conjugation gene clusters, *lacZ* gene fusions and β-galactosidase assays of thirteen and seven intergenic regions within the pUC11B and pNP40 conjugation gene clusters, respectively, were undertaken. These intergenic regions as identified within each of the two conjugation clusters were individually PCR-amplified and cloned upstream of the promoterless *lacZ* reporter gene of promoter probe vector pPTPL (O'Driscoll *et al*., 2004) in the case of the pNP40 intergenic regions, and in pPEPL for the pUC11B intergenic regions (oligonucleotides used are listed in Supplementary Table S3). The orientation and validity of the cloned intergenic regions were confirmed by restriction profile analysis and Sanger sequencing (Eurofins, Ebersberg, Germany). The application of the erythromycin resistance-conferring derivative of pPTPL, pPEPL, was required in this case as pUC11B harbors a tetracycline-resistance cassette, which would have impeded transformation/selection of the constructs into L. *cremoris* NZ9000 pUC11B.

Intergenic regions were named based on their downstream-located gene. For the pUC11B intergenic regions: U*trsA* (249 bps), U*trsR* (73 bps), U*trs07* (18 bps), U*trs09* (79 bps), U*trs10* (16 bps), U*trs12* (20 bps), U*trs13* (30 bps), U*trs15* (61 bps), U*trs19* (24 bps), U*trs20* (39 bps), U*trs22* (15 bps), U*trs24* (60 bps), U*trsAR* (245 bps). For pNP40: U*tra20* (752 bps), U*tra19* (95 bps), U*tra15* (97 bps), U*traL* (25 bps), U*traF* (103 bps), U*tra06* (99 bps), U*traA_a_* (208 bps). Oligonucleotides were designed to produce amplicons of ∼200 bps (Supplementary Table S3), with the start codon of the downstream-located gene as the reference point and located approximately in the midpoint of the amplicon, thus including sequence from downstream and upstream genes. The generated pPTPL/pPEPL plasmid constructs (nomenclature pPEPL::U*trsA* through to pPEPL::U*trsAR*, and pPTPL::U*tra20*-U*traA_a_*) were introduced into L. *cremoris* NZ9000 and L. *cremoris* NZ9000 with intact or mutated versions of either pNP40 or pUC11B, as required, by electrotransformation. The strains were grown overnight using standard conditions, and 200 µl of these overnight cultures were inoculated into 10 ml of GM17 and assayed for β-galactosidase activity, expressed in Miller Units (MU), as described previously (Miller, 1992) every 45 or 90 min, for a total of 405 min. As negative controls, L. *cremoris* NZ9000 pPTPL, NZ9000 pNP40 pPTPL or pUC11B pPEPL were tested, while strains harboring various pPTPL/pPEPL-derived constructs were assayed for β-galactosidase activity, which was calculated using the following formula: 1000 x [(OD_420nm_ - 1.75 x OD_550nm_)] / (T x V x OD_600nm_), where T represents the total time of the reaction in minutes and V represents the total volume of the culture, in mL, used in the assay. This was performed to assess the presence of active promoter sequences within the cloned regions, as indicated by a β-galactosidase-producing phenotype, and the possible regulatory effect that the presence of either the intact copies or the recombinant versions of either pNP40 or pUC11B would have in this activity.

### Fluorescence-based primer extension analysis

Fluorescence-based primer extension (FPE) was employed to detect transcriptional start sites (TSSs) within intergenic regions that displayed promoter activity in β-galactosidase assays. This was achieved using an established method (Schuster and Bertram, 2014), with the following modification. Total RNA from either L. *lactis* UC11 or L. *lactis* DRC3 was isolated and purified using the High Pure RNA isolation kit (Roche diagnostics) following the manufacturer's instructions, including an initial incubation step with 30 mg/mL of lysozyme at 37 ºC for 30 min to promote cell disruption.

Reverse transcription of this RNA was performed using specific oligonucleotides for each intergenic region with promoter activity, with a near-infrared dye attached at the 5′-end of the oligonucleotides, 5′ IRDye 700, purchased from Integrated DNA Technologies (IDT; Coralville, IA, USA). For reverse transcription, SuperScript III Reverse Transcriptase (Thermo-Fisher Scientific, USA) was used, following the manufacturer's instructions. Simultaneously, a Sanger sequencing reaction was performed using as template a PCR-amplified fragment of ∼1500 bps from either pUC11B or pNP40, with the relevant intergenic region sequence centrally located, and using the Thermo Sequenase Cycle Sequencing kit (Thermo-Fisher Scientific, USA) and the relevant IRD-labelled oligonucleotide. This resulted in a traditional Sanger sequencing ladder which, when compared to the length of the reverse transcribed DNA fragment obtained from the RNA, allowed mapping of the corresponding 5′ mRNA-based transcriptional start site. Separation of extension and sequencing products was achieved on 0.4 mm denaturing polyacrylamide gels consisting of 10% polyacrylamide (19:1 acrylamide:bis-acrylamide), 7 M urea and 1×TBE. Signal detection and image capture were performed with a 4300 DNA Analysis System (Li-COR Biosciences). Oligonucleotides employed for FPE are listed in Supplementary Table S3.

### Statistical data analysis

Data presented in this study are means ± standard deviation (SD) of triplicate assays. Obtained results were analysed using the SigmaPlot 11.0 statistical package (SPSS), from Systat Software, Inc., San Jose California USA. One-way analysis of variance ANOVA was performed to compare frequency of conjugation. A *P* value of ≤ 0.001 was considered significant and is represented with two asterisks “**” in the graphs.

## Results

### The conjugation clusters of pUC11B and pNP40 encompass multiple transcriptional units

**Fig. 1:**
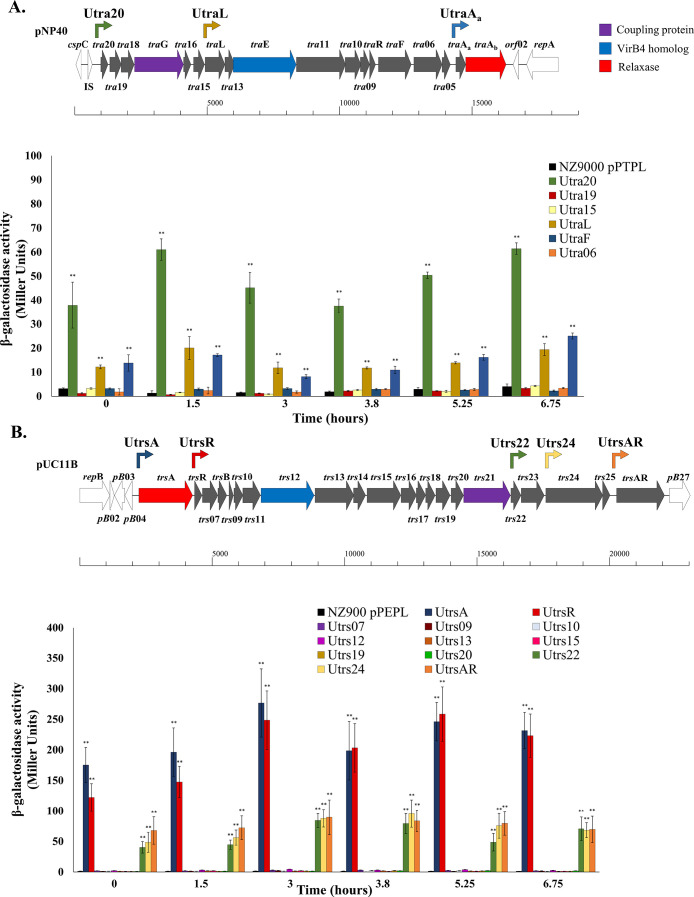
Time-course measurements of the β-galactosidase activity, expressed in Miller Units (MU), of: **(A)** seven L. *cremoris* NZ9000 pPTPL::Utra constructs and L. *cremoris* NZ9000 pPTPL as the negative control strain; **(B)** thirteen L. *cremoris* NZ9000 pPEPL::Utrs constructs and L. *cremoris* NZ9000 pPEPL as the negative control strain. In both cases, the associated conjugation gene clusters of either conjugative plasmid are indicated by dark-grey gene arrows, with the intergenic regions that displayed promoter activity marked by an arrow matching the colour of the bar graph below. The genes encoding the relaxase, the coupling and the VirB4 homolog were identified using CONJscan (Cury et al., 2020), and are color-coded in the gene graphs. All values were compared against the negative control, and a *P*-value ≤ 0.001 was considered significant and is represented by two asterisks “∗∗”. Presented data are the mean of three replicates ± standard deviation.

To determine whether the pNP40 and pUC11B conjugation gene clusters are each transcribed as a single operon, or consist of multiple transcriptional units, the presence of promoter activity provided by intergenic regions within these clusters was assessed. Intergenic regions were defined as the non-coding DNA sequences located within these conjugation gene clusters. Seven intergenic regions were identified within the pNP40 conjugation gene cluster and assigned names based on the downstream-located gene, i.e., U*tra20*, U*tra19*, U*tra15*, U*traL*, U*traF*, U*tra06*, U*traA_a_*, and similarly thirteen intergenic regions within the pUC11B conjugation cluster were identified, i.e., U*trsA*, U*trsR*, U*trs07*, U*trs09*, U*trs10*, U*trs12*, U*trs13*, U*trs15*, U*trs19*, U*trs20*, U*trs22*, U*trs24* and U*trsAR*. These regions were proposed as possible promoter-containing regions. To determine if any of these regions contained functional promoters, they were individually cloned upstream of the promoterless *lacZ* gene of pPTPL (in case of the pNP40 intergenic regions), or pPEPL (for pUC11B intergenic regions since pUC11B harbors a tetracycline-resistance cassette), and introduced into L. *cremoris* NZ9000, after which the β-galactosidase activity of these strains was determined. Time-course measurements (across 6.75 h of cultivation) of the β-galactosidase activity in L. *cremoris* NZ9000 pPTPL::U*tra* and pPEPL::U*trs* strains were annotated and compared (Fig. 1). L. *cremoris* NZ9000 harboring the empty pPTPL and pPEPL vectors were used as negative controls in the assays with associated β-galactosidase activity values of up to 2 MU.

Among the seven pNP40-derived intergenic regions, only U*tra20*, U*traL* and U*traA_a_* presented significant (*P* ≤ 0.001) differences compared to the negative control. These three intergenic regions were shown to contain active promoters, generating β-galactosidase activity ranging from 8.1 to 61.4 MU, with the first promoter displaying the highest activity (Fig. 1A). Among the thirteen evaluated pUC11B-derived intergenic regions, U*trsA*, U*trsR*, U*trs22*, U*trs24* and U*trsAR* were shown to result in significant (*P* ≤ 0.001) differences compared with the empty vector. The β-galactosidase activity promoted by these intergenic regions ranged from 40.3 to 276.6 MU, with the first two promoters displaying the highest activity (Fig. 1B). Under the conditions tested the β-galactosidase activity did not appear to be growth phase-dependent.

### Transcriptional start sites of the pNP40 and pUC11B conjugation gene clusters

**Fig. 2:**
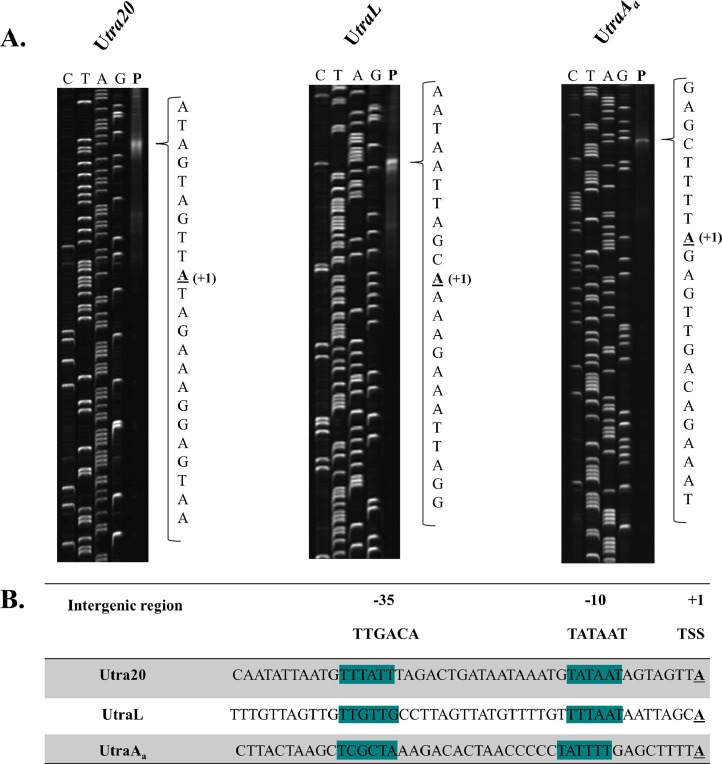
Primer extension analysis of the pNP40 conjugation gene cluster, tra. (**A)** Transcriptional start sites (TSSs) were determined using specific fluorescently labelled oligonucleotides. The corresponding DNA sequencing ladders (CTAG) were produced by the dideoxy chain termination method with the same primers and the primer extension products are indicated in the lanes marked **P**. The nucleotide sequence of the region of the TSS is indicated in each case, and the precise TSS is underlined. **(B)** Intergenic sequences highlighting the identified promoter-containing regions (−10 and −35; highlighted in teal) and transcription start sites (TSS).

**Fig. 3:**
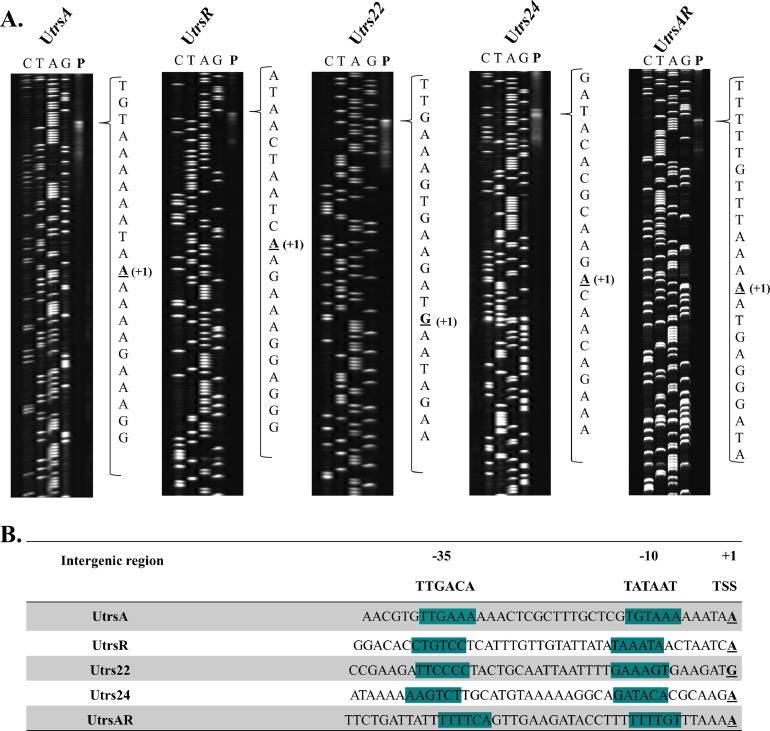
Primer extension analysis of the pUC11B conjugation gene cluster, trs. (**A)** TSSs were determined by primer extension, and the corresponding DNA sequencing ladders (CTAG) were produced by the dideoxy chain termination method with the same primers. The primer extension products are indicated in the lanes marked **P**. The nucleotide sequence of the region of the TSS is indicated in each case, and the precise TSS is underlined. **(B)** Intergenic sequences with identified promoter-containing regions (−10 and −35; highlighted in teal) and transcription start sites (TSS).

The intergenic regions that showed promoter activity in the conjugation gene clusters of pNP40 (U*tra20*, U*traL* and U*traA_a_*) and pUC11B (U*trsA*, U*trsR*, U*trs22*, U*trs24* and U*trsAR*) were further assessed by determining the precise location of their respective transcriptional start point. Based on a previous FPE protocol (Schuster and Bertram, 2014), eight different fluorescently labelled oligonucleotides were designed, each targeting one of the intergenic regions and annealing to the 5′ region of the relevant downstream gene (Supplementary Table S3). By means of this approach, the transcriptional start site (TSS) of each promoter region was determined. Within the pNP40 conjugation gene cluster (Fig. 2A), the 5′ end of the mRNA of the three identified promoters was mapped to an adenine nucleotide, 17, 24 and 31 nucleotides upstream of the presumed translational start site of *tra20, traL* and *traA_a_*, respectively. Similarly, the 5′ end of the mRNA of the five promoters located within the pUC11B conjugation gene cluster (Fig. 3A) was mapped to an adenine nucleotide, 23, 17, 23, and 15 nucleotides upstream of the predicted translational start site *trsA, trsR, trs24* and *trsAR*, respectively, and to a guanine nucleotide 21 nucleotides upstream of *trs22*. The −10 and −35 hexamer promoter consensus sequences of the mapped promoter-containing regions were manually identified based on their relative position to each of the identified TSSs and are presented in Figs. 2B and 3B.

**Fig. 4:**
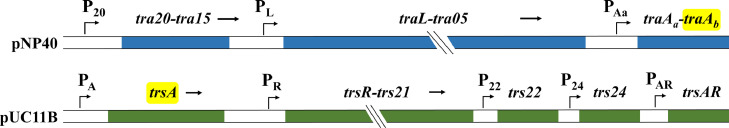
Organization of the pNP40 and pUC11B conjugation gene clusters. The pNP40 conjugation gene cluster (in blue) contains three transcriptional units, controlled by three promoters, P_20_, P_L_ and P_Aa_. The pUC11B conjugation gene cluster (green) starts with its relaxase-encoding gene trsA and contains five transcriptional units, which are controlled by five distinct promoters, P_A_, P_R_, P_22_, P_24_ and P_AR_. The relaxase-encoding genes are highlighted in yellow.

These results, paired with the *lacZ* gene fusions, validated the presence of three promoters (P_20_, P_L_ and P_Aa_) within the pNP40 conjugation gene cluster and five distinct promoters located within the pUC11B conjugation cluster (P_20_-P_Aa_) (Fig. 4). In both conjugative plasmids, the relaxase-encoding genes (*traA_b_* in the case of pNP40 and *trsA* for pUC11B) appear to be transcribed as a distinct unit, with the accessory factor TraA_a_ also being transcribed along with the relaxase TraA_b_ in the pNP40-encoded conjugation system.

### Plasmids pNP40 and pUC11B negatively regulate transcription of their conjugation machinery

**Fig. 5:**
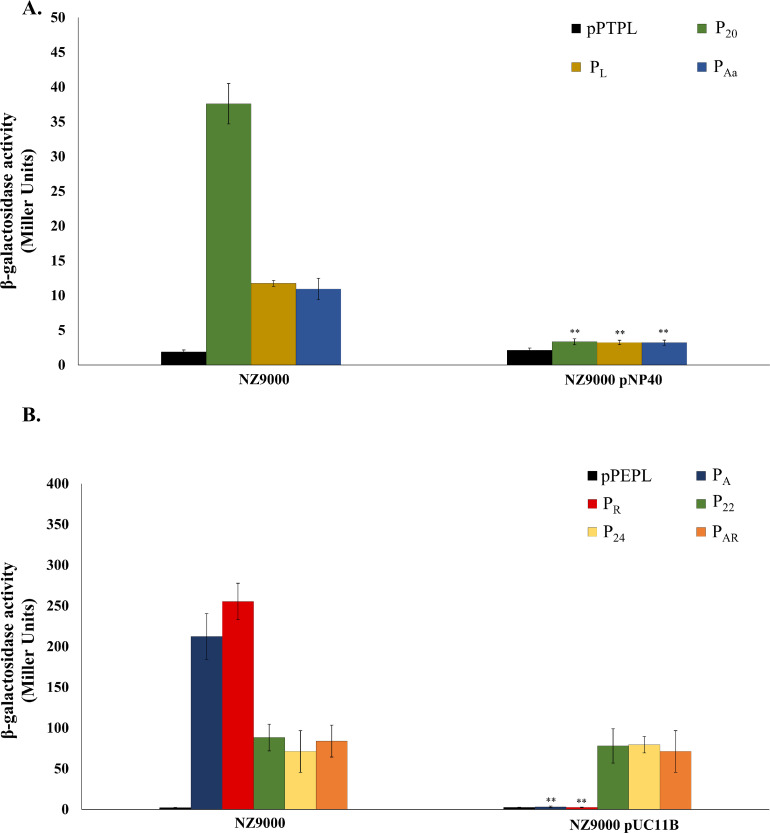
Quantification of the β-galactosidase activity, displayed as Miller Units (MU), of: **(A)** the three active promoter constructs from the pNP40 conjugation gene cluster, pPTPL::P_20_-P_L_-P_Aa_, in L. *cremoris* NZ9000 (control) and L. *cremoris* NZ9000 pNP40; **(B)** the five active promoter-containing constructs from the pUC11B conjugation cluster, pPEPL::P_A_-P_R_-P_22_-P_24_-P_AR_, introduced into L. *cremoris* NZ9000 (control) and L. *cremoris* NZ9000 pUC11B. All quantifications were made after 3.8 h of incubation, and similar results were obtained for the other time points 0, 1.5, 3, 5.25 and 6.75 h. Data was compared against the respective MU values in the absence of the conjugative plasmid, and a *P*-value ≤ 0.001 was considered very significant and is represented by two asterisks “∗∗”. Presented data are the mean of three replicates ± standard deviation.

We previously showed that the conjugation cluster present in either pNP40 or pUC11B encodes two and one putative repressors, respectively, belonging to the MerR-like (*tra20* and *trsR*) and H—NS-like (*traR*) families of transcriptional regulators (Ortiz Charneco *et al*., 2021, 2023). These studies had also demonstrated that a mutation in *traR* and *trsR* causes an increase in conjugation frequency, whereas no discernible effect on conjugation was observed when mutating *tra20*, under the conditions tested. Following the determination of the promoters located within the pNP40 and pUC11B conjugation gene clusters, further β-galactosidase assays were performed using the promoter fusion constructs in the presence of either pNP40 or pUC11B, respectively, to ascertain the impact of the conjugative plasmids on the activity of their conjugation gene cluster-associated promoters (Fig. 5). The three pPTPL::U*tra20*/U*traL*/U*traA_a_* constructs, which display promoter activity when present in L. *cremoris* NZ9000, significantly (*P* ≤ 0.001) reduced their β-galactosidase activity (to levels similar to that of the negative control) when pNP40 was present (Fig. 5A). Among the five pPEPL::U*trsA*/U*trsR*/U*trs22*/U*trs24*/U*trsAR* constructs that displayed promoter activity in L. *cremoris* NZ9000, only pPEPL::U*trsA* and pPEPL::U*trsR*, exhibited a significantly (*P* ≤ 0.001) reduced β-galactosidase activity in the presence of pUC11B (Fig. 5B). These results indicate that the presence of pNP40 exerts a significant negative effect on the activity of its three conjugation/related promoters, P_20_, P_L_ and P_Aa_, while pUC11B negatively regulates transcription of its conjugation gene cluster by acting on its first two promoters, P_A_ and P_R_.

**Fig. 6:**
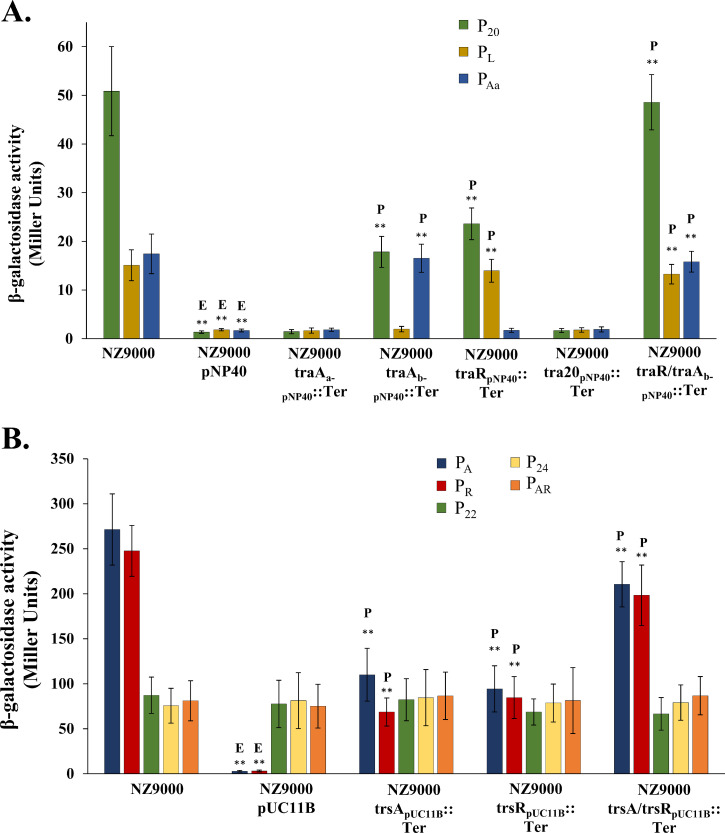
β-galactosidase activity assay, displayed as Miller Units (MU), of: **(A),** pPTPL::P_20_-P_L_-P_Aa_, in L. *cremoris* NZ9000, in the presence of wild type pNP40 or with the recombinant derivatives, *traA_a_*_-pNP40_::Ter, *traA_b-_*_pNP40_::Ter, *traR*_pNP40_::Ter, *tra20*_pNP40_::Ter or *traR*/*A_b_*_-pNP40_::Ter; **(B)** pPEPL::P_A_-P_R_-P_22_-P_24_-P_AR_ introduced into L. *cremoris* NZ9000, in the presence of wild type pUC11B or the recombinants *trsA*_pUC11B_::Ter, *trsR*_pUC11B_::Ter or *trsR*/*A*_pUC11B_::Ter. In both cases, the MU values of the strain with the conjugative plasmid were compared against the MU values from the control, L. *cremoris* NZ9000, in the absence of the conjugative plasmid, and a *P*-value ≤ 0.001 was considered very significant and is represented by two asterisks “∗∗” accompanied by the letter “E” (Empty strain). The MU values of the remaining strains harbouring the mutated versions of either pNP40 or pUC11B were compared against the MU values from the strains with unmutated conjugative plasmid, and a *P*-value ≤ 0.001 was considered very significant and is represented by two asterisks “∗∗” accompanied by the letter “P” (pNP40- or pUC11B-containing strain). Presented data are the mean of three replicates ± standard deviation.

To discern which elements within pNP40 and pUC11B are responsible for the observed regulatory effect, the impact of mutagenesis of specific genes within the conjugation cluster on the transcriptional fusions was assessed. The targeted genes were selected based on their predicted regulatory role via bioinformatic analysis and previous recombineering studies (Ortiz Charneco *et al*., 2021, 2023). The relaxase-encoding genes of pNP40 and pUC11B were also assessed for their regulatory role since it was shown that in the conjugative plasmid pIP501 from *Enterococcus faecalis* regulation of conjugation was mediated by the concerted action of both a transcriptional repressor and the relaxase (Kohler et al., 2018). Derivatives of pNP40 and pUC11B carrying mutations in individual genes were used to assess their effect on the identified promoters of the corresponding conjugation gene clusters using the pPTPL/pPEPL-based promoter fusions (see above). The selected candidate genes in pNP40 were *tra20, traR, traA_a_* and *traA_b_* (with one version of pNP40 including a double mutation in both *traR* and *traA_b_*), while the candidate genes in pUC11B were *trsA* and *trsR*, which were mutated individually and in combination. Mutations in *tra20* and *traA_a_* of pNP40 did not affect promoter activity relative to the unmutated pNP40 (Fig. 6A). However, mutation of *traR* caused an increase of promoter activity compared to the unmutated pNP40, suggesting that TraR plays a role in the regulation of the P_L_ promoter. Moreover, in the presence of this mutant, activity of P_20_ was significantly (*P* ≤ 0.001) increased, although the observed activity was not completely recovered to the same levels exhibited in the absence of pNP40, indicating that an additional element encoded by pNP40 is involved in the regulatory control of the system. Similarly, P_20_ displayed a very significant (*P* ≤ 0.001) increase in promoter activity in the presence of the pNP40 *traA_b_* mutant, compared to the complete repression observed in the presence of the wild type pNP40, but this activity was not completely recovered to the same levels displayed by this construct in the absence of the conjugative plasmid. The P_Aa_ promoter displayed a very significant (*P* ≤ 0.001) increase in its activity in the presence of the *traA_b_* mutant of pNP40, similar to its activity in the absence of pNP40. Finally, the promoter activity of P_20_ in the presence of pNP40 in which both genes *traR* and *traA_b_* had been mutated was the same as that in the absence of pNP40, suggesting that the products of both genes operate in concert to control activity of the first promoter (P_20_) of the pNP40 conjugation gene cluster.

For the pUC11B system, mutations in either *trsA* or *trsR* were shown to cause an increase in the activity of promoters P_A_ and P_R_ (Fig. 6B), compared to the unmutated pUC11B. Furthermore, promoter activity of these two promoters in the presence of the double mutant derivative of pUC11B, *trsA/trsR*_pUC11B_, was similar to that observed in the absence of pUC11B, suggesting that the products of *trsA* and *trsR* regulate expression of the P_A_ and P_R_ promoters of the pUC11B conjugation gene cluster.

### Specific binding of relaxases and repressors to the promoter regions

Based on the β-galactosidase assay results presented above, the presumed relaxases of pNP40 and pUC11B, TraA_b_ and TrsA, respectively, along with their predicted respective repressor proteins TraR and TrsR (Ortiz Charneco *et al*., 2021, 2023), were determined to play a role in regulating transcription of the conjugation gene clusters. To assess if the above-mentioned proteins directly interact with the promoters they regulate we performed DNA-protein binding studies employing EMSAs. These four proteins, along with the accessory factor TraA_a_ and the predicted transcriptional repressor of the pNP40 conjugation cluster, Tra20, were overexpressed and purified. Despite not presenting any significant effects in regulating promoter activity in pNP40, Tra20 was selected for this approach due to its high structural similarity to the transcriptional repressor of the pIP501-encoded conjugation system TraN (Grohmann et al., 2016; Ortiz Charneco *et al*., 2021), as well as with the pNP40-encoded TraR. Fluorescently labelled PCR fragments encompassing each promoter sequence were used to assess specific binding of the proteins to these regions.

**Fig. 7:**
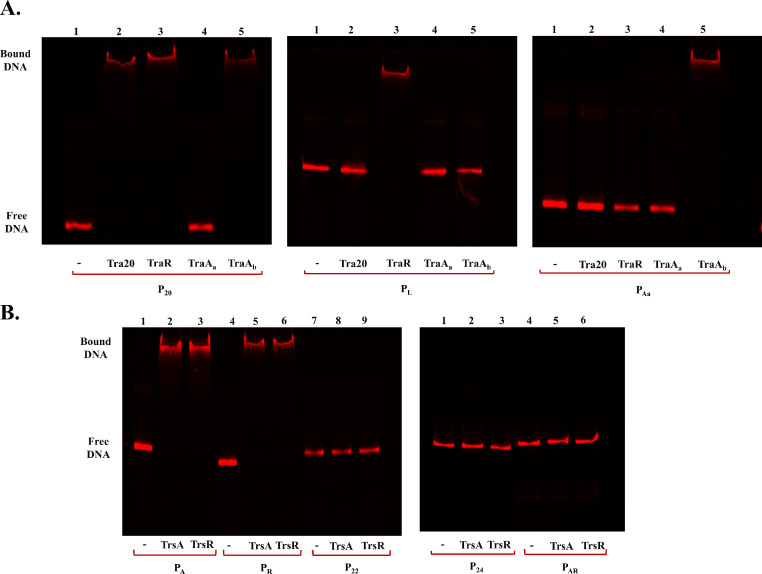
EMSA gel images pertaining to: **(A)** PCR fragments (0.1 pmol) containing the sequences of the three promoters of the pNP40 conjugation gene cluster, P_20_, P_L_ and P_Aa_, each tested for specific protein binding with 250 nM of either Tra20 (lane 2), TraR (lane 3), TraA_a_ (lane 4) or TraA_b_ (lane 5). The first lane corresponds to the IRD700-labelled PCR fragment in the absence of protein; **(B)** PCR fragments (0.1 pmol) containing the five promoter-sequences of the pUC11B conjugation gene cluster, P_A_, P_R_, P_22_, P_24_ and P_AR_, each assayed for specific protein binding with 250 nM of either TrsA (lanes 2, 5 and 8) or TrsR (lanes 3, 6 and 9). Lanes 1, 4 and 7 correspond to the IRD700-labelled PCR fragment in the absence of protein. Band shift represents protein binding.

PCR fragments containing the three different promoters from the pNP40 conjugation gene cluster were tested for binding by the repressor and relaxase proteins Tra20, TraR, TraA_a_ and TraA_b_ (Fig. 7A). Proteins TraR and TraA_b_ were shown to specifically bind to the DNA sequence containing the P_20_ promoter, corroborating the results obtained in the β-galactosidase assays. Tra20 also bound specifically to the PCR fragment containing P_20_, suggesting it plays a role in regulating this promoter, though perhaps under conditions that are distinct from the in vivo conditions tested here. TraR was capable of binding to the second promoter-containing fragment, P_L_, and TraA_b_ bound to the third promoter-containing fragment, P_Aa_. Following the same approach for the pUC11B conjugation-related promoters (Fig. 7B), both TrsA and TrsR were shown to specifically bind to the PCR fragments containing the first and second promoters, P_A_ and P_R_, respectively, with no observable binding to any of the remaining three promoters, thereby validating our previously obtained results (the effect of increasing protein concentrations in protein binding can be observed in Supplementary Fig. S1). With the exception of Tra20, in each case where a particular protein bound to a specific promoter the binding corresponded to the promoter activity being affected by the mutation of the gene encoding that binding protein (Fig. 6).

### Transcriptional regulation occurs in the vicinity of the −35/−10 promoter sequences

**Fig. 8:**
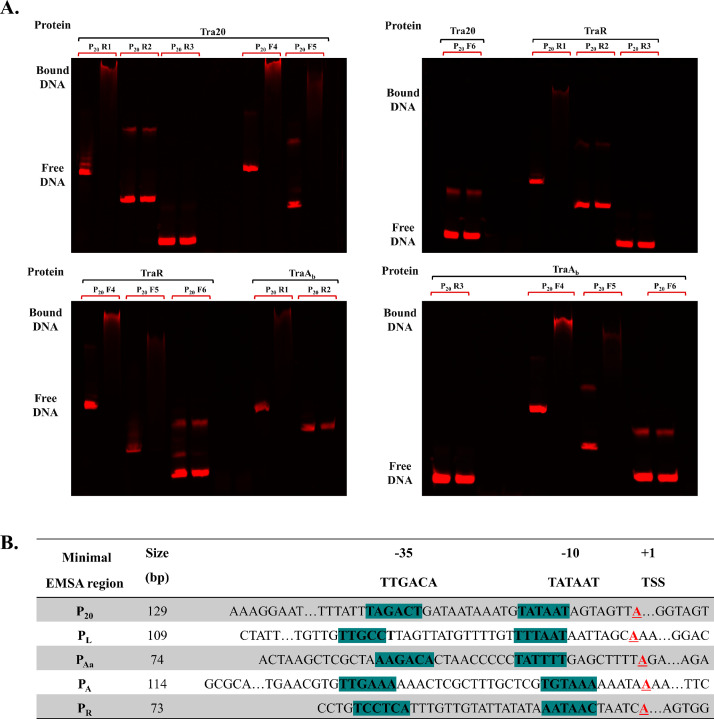
**(A)** EMSA gel images using 250 nM purified Tra20, TraR or TraA_b_ and 0.1 pmol of differently sized of IRD700-labelled PCR fragments located close to P_20_ of pNP40. **(B)** Minimal sequences required for specific protein binding to each promoter-containing region, based on specific protein binding to each fragment. In all cases, the −35 and −10 promoter consensus boxes, as well as the TSS, is present in the sequence. Gel images displaying specific protein binding to each PCR fragment is available in Supplementary Figure S2.

The specific binding of the presumed nickases of pNP40 and pUC11B (TraA_b_ and TrsA), and the predicted transcriptional regulators (TraR, TrsR and Tra20) was demonstrated although the precise DNA sequence they bind to is unknown. Therefore, further assays were performed to determine the minimal sequence within each promoter-containing region required for protein binding. For this purpose, successively smaller fragments of the promoter regions were applied in EMSAs with these five proteins (Fig. 8A, Supplementary Table S4). Proteins specifically bound to several fluorescently labelled PCR-derived fragments, depending on their sizes, and the precise location and sizes of these fragments, as well the band shifts, can be observed in detail in the supplemental material (Supplementary Table S4 and Supplementary Fig. S2). Through this approach, it was established that both TraA_b_ and TraR specifically bind to the assigned −35 and −10 promoter sequences of P_20_, P_L_ and P_Aa_, and similarly TrsA and TrsR specifically bound to PCR fragments including the designated −35 and −10 promoter sequences of P_A_ and P_R_ (Fig. 8B)..

## Discussion

Conjugation systems in Gram-positive bacteria are significantly less characterized than their counterparts in Gram-negative bacteria, despite notable efforts in recent years in *Streptococcus thermophilus* (Cappele et al., 2021), *B. subtilis* (Mori et al., 2021) and *S. aureus* (Yui Eto *et al*., 2021), among others (Goessweiner-Mohr et al., 2014; Kohler et al., 2019; Miguel-Arribas et al., 2022). In particular, conjugation among lactococcal strains remains a subject of great interest to the dairy industry, which have widely employed plasmid conjugation to enhance the robustness of starter strains (Mills et al., 2006; Ainsworth et al., 2014).

To date, the transcriptional regulation of conjugation in lactococci has not been investigated and based on our findings, conjugation efficiency appears to be highly dependent on the assay conditions. The naturally occurring conjugative plasmids pNP40 and pUC11B each harbor one of the two prevalent types of conjugation systems among lactococci, and have been subject to recent characterization efforts, which highlighted the presence of a putative transcriptional repressor encoded by a gene present in each conjugation gene cluster (Ortiz Charneco *et al*., 2021, 2023). The relaxase class of both plasmids was bioinformatically determined using MOBscan (Garcillán-Barcia et al., 2020), with traA_b_ from pNP40 being member of the MOB_P_ family of relaxases, while trsA from pUC11B representing a member of the MOB_Q_ family of relaxases. Both relaxase families belong to the HUH endonuclease family, which are characterized by their conserved two-histidine motif (Lucas and Moncalián, 2020). Additionally, CONJscan (Cury et al., 2020) was used to determine the mating pair formation (MPF) system of both plasmids, which are the Type 4 Secretion System (T4SS) proteins that constitute the *trans*-envelope transport channel (Goessweiner-Mohr et al., 2014). There are currently two phylogenetically different MPF systems recognized in Firmicutes, MPF_FA_ and MPF_FATA_ (Guglielmini et al., 2013). The conjugative plasmids pNP40 and pUC11B are predicted to be both representatives of the MPF_FATA_ system.

In the present study, we report on the identification of three and five active promoter-containing regions within the pNP40 and pUC11B conjugation gene clusters, respectively. It is noteworthy that despite representing two divergent conjugation systems among lactococcal plasmids, both pNP40- and pUC11B-encoded relaxases appear to be transcribed separately from the rest of the conjugation gene cluster, despite the relaxase-encoding gene from pUC11B being located at the 5′ end of the cluster and the relaxase-encoding gene from pNP40 being present at the 3′ end of its conjugation cluster. This is in contrast to other conjugation systems, such as those encoded by pIP501 in *E. faecalis* and pLS20 in *B. subtilis*, in which the relaxosome-related genes are not located close to the origin of replication and have been reported to be embedded with the rest of the conjugation genes and under the common control of the main conjugation promoter (Miguel-Arribas et al., 2017; Kohler et al., 2018). With respect to pUC11B, it has previously been reported that mutation of any of the last five genes of the conjugation cluster, i.e. *trs22, trs23, trs24, trs25* and *trsAR,* has no impact on conjugation frequency (Ortiz Charneco *et al*., 2023). Herein these genes have been reported to be transcribed separately from the main conjugation operon, which suggests that the functional role these genes play during a pUC11B-mediated conjugation event is not required for conjugation to occur under the conditions tested. Moreover, despite their initial identification via *lacZ* gene fusions and subsequent mapping, not all promoter-containing regions exhibit clear −10 and −35 promoter consensus sequences, as presented in Fig. 3B. Particularly those upstream of *trs22, trs24* and *trsAR* displayed a poorly conserved −10 sequence. Interestingly these promoter-containing regions also exhibited significantly lower β-galactosidase activity compared to P_A_ and P_R_. Nevertheless, despite *trs22, trs23, trs24, trs25* and *trsAR* not being apparently required for conjugation (Ortiz Charneco *et al*., 2023), they seem to be relatively well conserved among pUC11B-like conjugation systems. Moreover, the *trsAR* gene has been shown to play a role in overcoming type II and type III restriction-modification (R/M) systems in the recipient cell, thus broadening the host range of the plasmid (Ortiz Charneco *et al*., 2023).

Overall, regulation of conjugation on plasmids pNP40 and pUC11B appears to be reminiscent of that observed in other conjugation systems from various Gram-positive organisms. Both lactococcal systems are tightly controlled by proteins encoded within their conjugation clusters, as is the case of plasmid pLS20 from *B. subtilis*, which presents a conjugation system with a promoter that is repressed in a complex manner by several proteins encoded in the same plasmid (Singh et al., 2020). Similarly, the relaxase from the promiscuous streptococcal plasmid pMV158, MobM, not only acts as a transcriptional repressor of its own gene (Lorenzo-Díaz et al., 2012), but of the antisense RNA, thus regulating plasmid copy number and increasing the plasmid molecules that are horizontally and/or vertically transferred (Lorenzo-Díaz et al., 2017). Another significant similarity transpires when comparing the proposed model for the regulation of conjugation in plasmids pNP40 and pUC11B to that of the *E. faecalis* model conjugative plasmid, pIP501. We report that the conjugation promoters of plasmids pNP40 (P_20_) and pUC11B (P_A_ and P_R_) are negatively (auto)regulated by a concerted action between their respective relaxases and repressor proteins. Transcription of the pIP501 conjugation gene cluster is governed by two promoters (P_tra_ and P_traNO_), and the transcription of the promoter controlling the expression of the majority of the *tra* genes, P_tra_, is repressed by the conjoined action of the relaxase, TraA and the repressor TraN, which in turn also regulates its own production and that of the surface adhesin of the system, TraO (Kohler et al., 2018). In the present study, the binding regions of the relaxases and conjugative repressors of the pNP40 and pUC11B conjugation gene clusters were delineated and were shown to include predicted −35 and −10 promoter sequences. The relaxase TraA in pUC11B and TraA in pIP501 have similar autoregulation, where in both systems the relaxase-encoding genes are located in the 5′-end of the conjugation gene cluster. By specifically binding to an area that overlaps with the −10 region of the first promoter of its conjugation cluster, the relaxase's own expression is driven (Kurenbach et al., 2006). A similar binding pattern is proposed in this study for the relaxases of plasmids pNP40 and pUC11B, although the determination of the specific binding site, at the nucleotide level, of the relaxases and conjugative repressors of these conjugation systems remains a subject for future studies.

**Fig. 9:**
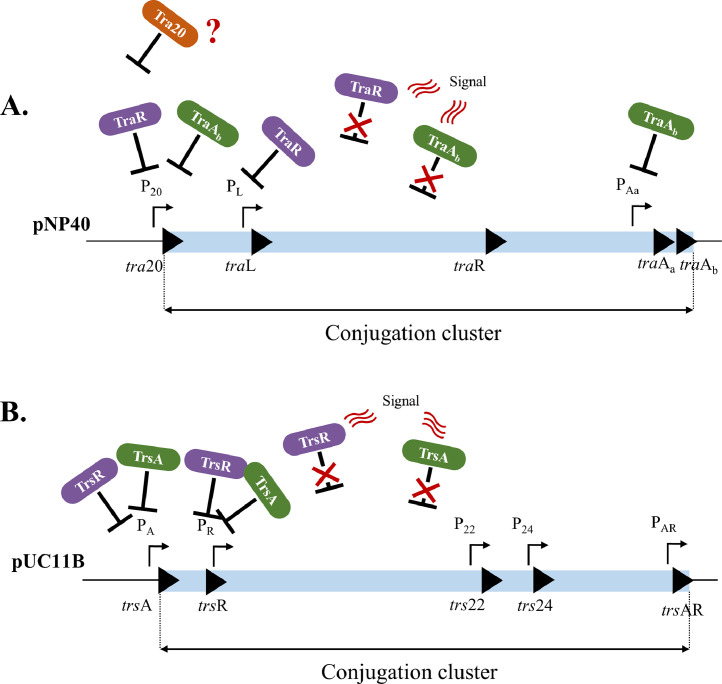
Schematic representation of the: **(A)** regulation of the pNP40 conjugation cluster. The relaxase TraA_b_ regulates its own expression by binding to the third promoter (P_Aa_) upstream traA_a_, while also regulating the first promoter (P_20_) of the cluster, in conjoined action with TraR, which in turn regulates the transcription of the second promoter (P_L_) of the conjugation cluster. The protein Tra20 is suggested to play an as of yet undetermined role in the regulation of the pNP40 conjugation gene cluster; **(B)** regulation of the pUC11B conjugation gene cluster, with both the relaxase TrsA and the repressor TrsR regulating the transcription of the two first promoters of the cluster, P_A_ and P_R_. In both systems, the as of yet unidentified signals responsible for relief of transcriptional repression by these proteins are indicated by waving red lines.

Based on the results of the β-galactosidase and EMSA assays, a working model of the regulation of the pNP40- (Fig. 9A) and pUC11B-encoded (Fig. 9B) conjugation gene clusters was generated, including the as yet hypothetical signal that is hereby suggested to be involved in the relief of transcriptional repression of the gene clusters. During pLS20-induced conjugation, this signal has been reported to be quorum-sensing-related (Mori et al., 2021), but no such mechanism has yet been proposed for either pNP40 or pUC11B conjugation systems. The relaxases of both pNP40 and pUC11B appear to present two distinct binding activities: they are responsible for initiation of the conjugation process by introducing a ssDNA break at the *oriT* sequence of their respective plasmids in an, as yet, undetermined manner, while also being involved in the transcriptional regulation of several conjugation-related promoters. Finally, the relaxases TraA_b_ and TrsA also appear to regulate their own expression in what seems to represent an autoregulatory loop.

Additionally, specific binding of Tra20 to the first promoter of the pNP40 conjugation gene cluster, P_20_, was observed. The gene encoding this protein was reported to have no effect on conjugation frequency when mutated (Ortiz Charneco *et al*., 2021), nor had it any effect on the activity of the promoters of the pNP40 conjugation cluster. Nonetheless, this protein displays significant structural similarities to the MerR family of transcriptional repressors (Ortiz Charneco *et al*., 2021), as do TraR of pNP40 and TraN, the transcriptional repressor of the pIP501 conjugation system (Kohler et al., 2019). Despite not observing any regulation effect mediated by Tra20, its specific binding to P_20_ suggests that it acts as a regulator although the conditions under which this occurs remain to be defined.

This report signifies a comprehensive analysis on the transcription of pNP40 and pUC11B conjugation gene clusters, and how this transcription is regulated by elements located within these clusters. This regulation appears to be a repression of these promoter-containing regions, mediated by the specific binding of the relaxases and the transcriptional repressor of these clusters. The signals that are detected by these proteins and promote their detachment from their respective promoter-containing regions, remains unidentified. Recent studies have identified a quorum-sensing peptide encoded by the conjugative plasmid pLS20 in *B. subtilis* that is responsible for binding to the repressor of its conjugation cluster, promoting a conformational change in this protein and inactivating it, thus significantly increasing conjugation (Meijer et al., 2021). However, it remains subject to future studies to identify the signals responsible for the deactivation of the repression activity in the conjugative plasmids pNP40 and pUC11B. The potential discovery of such signals and/or any peptides involved in the deactivation of such repression could signify a major breakthrough that would significantly enhance conjugation processes among lactococcal strains facilitating the rapid enhancement of existing strain repertoires and strain robustness in the industrial setting.

## Funding

This publication has emanated from research conducted with the financial support of Science Foundation Ireland under Grant Numbers 12/RC/2273 and 17/SP/4678, which is co-funded by dsm-firmenich, Taste, Texture & Health.

## CRediT authorship contribution statement

**Guillermo Ortiz Charneco:** Conceptualization, Methodology, Validation, Formal analysis, Investigation, Writing – original draft, Writing – review & editing, Visualization. **Philip Kelleher:** Conceptualization, Software. **Andrius Buivydas:** Conceptualization. **Paul P. de Waal:** Conceptualization, Writing – review & editing, Funding acquisition. **Irma M.H. van Rijswijck:** Conceptualization, Writing – review & editing, Funding acquisition. **Noël N.M.E. van Peij:** Conceptualization, Writing – review & editing, Funding acquisition. **Jennifer Mahony:** Conceptualization, Validation, Writing – review & editing, Supervision, Project administration, Funding acquisition. **Douwe Van Sinderen:** Conceptualization, Validation, Writing – review & editing, Supervision, Project administration, Funding acquisition.

## Declaration of competing interest

PdeW, IvR & NvP are employed by the company dsm-firmenich. The remaining authors declare that the research was conducted in the absence of any commercial or financial relationships that could be construed as a potential conflict of interest.

## Data Availability

The data pertaining to the complete sequences of plasmids pNP40 and pUC11B is available in the NCBI Genbank repository, accession numbers NC_010901.1 and NZ_CP016721.3, respectively.

## Appendix Supplementary materials

Image, application 1

Image, application 2

Image, application 3

Image, application 4

Image, application 5
