# Supplementary material for: Transcriptional control of two distinct lactococcal plasmid-encoded conjugation systems
Source: Curr Res Microb Sci. 2024 Feb 5;6:100224. doi: 10.1016/j.crmicr.2024.100224 (PMC10873654; doi:10.1016/j.crmicr.2024.100224)
Supplement: Supplementary file 1 [file mmc1.docx]

**Supplementary Table S1.** Lactococcal strains used in this study.

| **Strain** | **Plasmids present in the strain** | **Relevant properties** |
| --- | --- | --- |
| **L. cremoris NZ9000 pNP40-pUC11B** | pNP40-pUC11B | Two separate strains harboring either the conjugative plasmid pNP40 (*nisR*) or the conjugative plasmid pUC11B (*tetR*) |
| **L. cremoris MG1614** |  | Main recipient strain, plasmid-free and streptomycin resistant |
| **L. cremoris NZ9000** |  | Derivative of the parental strain *L. cremoris* MG1363 |
| **L. cremoris NZ9000 tra_pNP40_, pJP005** | *tra20-traR-traA_a-_*_pNP40_, pJP005 | Strains harboring plasmid pJP005, *Cm^R^* and encoding *RecT*, as well as one of the three mutants of plasmid pNP40 |
| **L. cremoris NZ9000 traR/traA_b-pNP40_, pJP005** | *traR/traA_b-_*_pNP40_, pJP005 | Strain harboring plasmid pJP005, *Cm^R^*, and the double mutant of plasmid pNP40 |
| **L. cremoris NZ9000 trsR_pUC11B_, pJP005** | *trsR*_pUC11B_, pJP005 | Strain harboring plasmid pJP005, *Cm^R^* and encoding *RecT*, and the *trsR* mutant of plasmid pUC11B |
| **L. cremoris NZ9000 trsA/trsR_pUC11B_, pJP005** | *trsA/trsR*_pUC11B_, pJP005 | Strain harboring plasmid pJP005, *Cm^R^*, and the double mutant of plasmid pUC11B |
