# Supplementary material for: Transcriptional control of two distinct lactococcal plasmid-encoded conjugation systems
Source: Curr Res Microb Sci. 2024 Feb 5;6:100224. doi: 10.1016/j.crmicr.2024.100224 (PMC10873654; doi:10.1016/j.crmicr.2024.100224)
Supplement: Supplementary file 2 [file mmc2.docx]

**Supplementary Table S2.** Plasmids and plasmid constructs employed in this study.

| **Plasmid** | **Characteristics** | **Reference** |
| --- | --- | --- |
| **pNZ44E** | Erythromycin-resistant, *E. coli*-*L. lactis* shuttle vector pNZ44, P44 constitutive promoter | Draper *et al*., 2009 |
| **pNZ8048E** | Erythromycin-resistant version of the high-copy-number *E. coli*-*L. lactis* overexpression vector, P*nisA;* Erm^r^ | This study |
| **pNZ8048E::tra20-A_b_** | pNZ8048E derivatives containing one of the four genes selected within the pNP40 conjugation gene cluster | This study |
| **pNZ8048E::trsA-R** | pNZ8048E derivatives containing one of the two genes selected within the pUC11B conjugation gene cluster | This study |
| **pPTPL** | pPTP derivative and promoter-screening vector, which contains a promoterless *lacZ* gene and tetracycline resistance | O’Driscoll *et al*., 2004 |
| **pPEPL** | pPTPL derivative and promoter-screening vector, which contains a promoterless *lacZ* gene and erythromycin resistance | This study |
| **pPTPL::Utra20-A_a_** | pPTPL derivatives containing one of the 7 intergenic regions present within the pNP40 conjugation cluster | This study |
| **pPEPL::UtrsA-RM** | pPEPL derivatives containing one of the 13 intergenic regions present within the pUC11B conjugation cluster | This study |
