# Supplementary material for: Transcriptional control of two distinct lactococcal plasmid-encoded conjugation systems
Source: Curr Res Microb Sci. 2024 Feb 5;6:100224. doi: 10.1016/j.crmicr.2024.100224 (PMC10873654; doi:10.1016/j.crmicr.2024.100224)
Supplement: Supplementary file 3 [file mmc3.docx]

**Supplementary Table S3.** Oligonucleotides used in this study.

| Oligonucleotide name | Oligonucleotide sequence (5’ → 3’) |
| --- | --- |

| Recombineering primers^ᴪ^ |  |
| --- | --- |
| *trsA*_pUC11B_::Ter | G*C*A*T*T*GCTTGGTTTTAAAATAAAAGTTTTAGGAGCAACACTTCAACTCTAGAAATTAAATTTGTTATAGCGTTCACTGTATAATTTTTCC |
| *traA_b_*_-pNP40_::Ter | T*G*T*A*A*AACCATGAAAGAATATCTTGGAAATTCTGCGGAGTGAATTCTTTAAATTATCGCCACTCATACTGATAAAGACCACCTGCATAAT |
|  |  |
| Recombineering screening |  |
| ScrntrsA-Fw | GTTGATGAACAGCATTTTTTGAA |
| ScrntrsA-MAMA-Fw | CTATAACAAATTTAATTTCTAGAGTTG |
| ScrntrsA-Rv | CCAATTTTCACGCCATTGT |
| ScrntraA_b_-Fw | CTTCGACAAAATCTGAACTCAAACGTA |
| ScrntraA_b_-MAMAFw | GCGGAGTGAATTCTTTAAATTATC |
| ScrntraA_b_-Rv | TCAACACAGCGTGCAGGTATCTTAATC |
|  |  |
| pPEPL assembly^ᴪ^ |  |
| pPTPL-Fw | AAAAAATGTACAGGTTTAGAACTATGAGTGGCTAG |
| pPTPL-Rv | AAAAAACTCGAGGACTTATTGTCAGATCTAATCG |
| Ery-Fw | AAAAAACTCGAGACCAAGACGAAGAG |
| Ery-Rv | AAAAAATGTACAGACATACTGTTCTTCC |
|  |  |
| pNZ8048E assembly^ᴪ^ |  |
| pNZ8048-Fw | AAAAAATGTACATATGAGATAATGCCGACTGTACT |
| pNZ8048-Rv | AAAAAAGAATTCTTGATATGCCTCCTAAATTTTTATC |
| Ery-Fw | AAAAAAGAATTCACCAAGACGAAGAG |
| Ery-Rv | AAAAAATGTACAGACATACTGTTCTTCC |
|  |  |
| Protein overexpression primers^ᴪ ₳^ |  |
| Tra20-Fw | AAAAAACCATGGAACATCACCATCACCATCACGAAAATAATTGGTATAC |
| Tra20-Rv | AAAAAAGAGCTCATTAGCCTTAAAAAATGTTAAGA |
| TraR-Fw | AAAAAACCATGGAACATCACCATCACCATCACAACCAAACCTTACAAG |
| TraR-Rv | AAAAAAGAGCTCAAGCTAGTTACTGATAATGATTTT |
| TraA_a_-Fw | AAAAAACCATGGATGAAAAAAATCAAAAATCGTGAA |
| TraA_a_-Rv | AAAAAAGAGCTCTTAGTGATGGTGATGGTGATGCTCCCCCTCTGAAATAAA |
| TraA_b_-Fw | AAAAAACCATGGATGACAGTCATTAAAATGCCAAA |
| TraA_b_-Rv | AAAAAAGAGCTCCTAGTGATGGTGATGGTGATGAATTTCAAAGTCATCATCAC |
| TrsA-Fw | AAAAAACCATGGATGACTATTTATCATTTTGAAGCT |
| TrsA-Rv | AAAAAAGAGCTCTTAGTGATGGTGATGGTGATGCATTTTTGGACTGTATCCT |
| TrsR-Fw | AAAAAACCATGGCTCATCACCATCACCATCACATTTATCATTTTGAAGCT |
| TrsR-Rv | AAAAAAGAGCTCTTACATTTTTGGACTGTATCCT |
|  |  |
| pPTPL/pPEPL cloning primers for promoter mapping^ᴪ^ |  |
| pPEPL::UtrsA-Fw | AAAAAAGAATTCCTTCCTAGTCCTTTCTTATTTG |
| pPEPL::UtrsA-Rv | AAAAAAGTCGACGACTGTCCTTTCCCTCTTG |
| pPEPL::UtrsR-Fw | AAAAAAGAATTCTAGAGGATACAGTCCAAAAATG |
| pPEPL::UtrsR-Rv | AAAAAAGTCGACCAAGCTAAACCACTTATCTTTC |
| pPEPL::Utrs07-Fw | AAAAAAGAATTCACCGCCTAAGAATCCTACC |
| pPEPL::Utrs07-Rv | AAAAAAGTCGACGGTTTCCCCTGTTTCAAATG |
| pPEPL::Utrs09-Fw | AAAAAAGAATTCGGCTCTACGGATTGTTCAC |
| pPEPL::Utrs09-Rv | AAAAAAGTCGACTTTTAAAACTCCGCTATCTAGC |
| pPEPL::Utrs10-Fw | AAAAAAGAATTCAAATTAGAGAAGAAGGGCTATG |
| pPEPL::Utrs10-Rv | AAAAAAGTCGACCAACGCCATATTTATTTGTGAC |
| pPEPL::Utrs12-Fw | AAAAAAGAATTCCAAATTAGAGGCATTACAAAGG |
| pPEPL::Utrs12-Rv | AAAAAAGTCGACTTTTTTCATAGACGGAAGTAGG |
| pPEPL::Utrs13-Fw | AAAAAAGAATTCTATTCTACAGGTGAACCTATC |
| pPEPL::Utrs13-Rv | AAAAAAGTCGACGTGATTGGTGCGCTAATTC |
| pPEPL::Utrs15-Fw | AAAAAAGAATTCATTGACGACAAAGACCTTCC |
| pPEPL::Utrs15-Rv | AAAAAAGTCGACGAATGAATAATCCTGCTACAAG |
| pPEPL::Utrs19-Fw | AAAAAAGAATTCGCGCAAAAAAACTCTACGAAG |
| pPEPL::Utrs19-Rv | AAAAAAGTCGACAGAGAAATGCTATTACTCCTAAG |
| pPEPL::Utrs20-Fw | AAAAAAGAATTCGGCGCAGTTAAAAAAGTACG |
| pPEPL::Utrs20-Rv | AAAAAAGTCGACTTGCATAGACAATAAGTTTTCTC |
| pPEPL::Utrs22-Fw | AAAAAAGAATTCGAAAACCGTTCATATTCTGAC |
| pPEPL::Utrs22-Rv | AAAAAAGTCGACAAACATACAAGGACAATTAGAAG |
| pPEPL::Utrs24-Fw | AAAAAAGAATTCTTTCCAACGCTACTCGAATG |
| pPEPL::Utrs24-Rv | AAAAAAGTCGACGGCTTGATTGGGTTTTTCTG |
| pPEPL::UtrsAR-Fw | AAAAAAGAATTCCCCGAATATAAAGCCCTTTATG |
| pPEPL::UtrsAR-Rv | AAAAAAGTCGACGCACTTTTTTTACGTCTATAGTAT |
| pPTPL::Utra20-Fw | AAAAAAGAATTCACAACGATTAAGTTATGAACAAAA |
| pPTPL::Utra20-Rv | AAAAAAGTCGACTGTTTACTCCTTTCTATAACTACT |
| pPTPL::Utra19-Fw | AGCACAGAATTCGGCTAATAGCCTTAAAAAA |
| pPTPL::Utra19-Rv | ATCATAGTCGACACTTACCACTTCTTTCA |
| pPTPL::Utra15-Fw | AGCACGGAATTCTATTAAAAAAATAGCTTTCGGG |
| pPTPL::Utra15-Rv | AGCAGCGTCGACATTTTTTGCCTTTCTTTTTTG |
| pPTPL::UtraL-Fw | AAAAAAGAATTCTTCGGTAGCTTTAATTACTGG |
| pPTPL::UtraL-Rv | AAAAAAGTCGACCCTACTTTAACAGTTGAACTAG |
| pPTPL::UtraF-Fw | AGCAGCGAATTCCTAGCTTTAGATAACTAAT |
| pPTPL::UtraF-Rv | AGCAGGGTCGACTTTAAACCTCCTTATAGT |
| pPTPL::Utra06-Fw | AGCAGCGAATTCCATGATGAAAAAGTTATCC |
| pPTPL::Utra06-Rv | AGCATAGTCGACGTTTTCCCTACTTTCAT |
| pPTPL::UtraA_a_-Fw | AAAAAAGAATTCACCGAAGAGGAAGTTAAAAATT |
| pPTPL::UtraA_a_-Rv | AAAAAAGTCGACCTTGTCAACCTCTCAAATTTC |
|  |  |
| Template primer extension primers |  |
| TemptrsA-Fw | CGTTTTTGTTGTCCAATTTTTC |
| TemptrsA-Rv | ATTTGTTCCGTTTCTTTTAAGTC |
| TemptrsR-Fw | GCTTTCCAAGGCATTATCC |
| TemptrsR-Rv | CTGTTCTTGTTTAGGGACTTC |
| Temptrs22-Fw | GATATGGCATTGGTGTAGG |
| Temptrs22-Rv | CCGCCTATCGCTAACTC |
| Temptrs24-Fw | AATTATTGTCGGTATGTTTTCC |
| Temptrs24-Rv | GTGTTGCTGTAGAAAATCATC |
| TemptrsAR-Fw | CAGACCTATTAAGTGGCAAAAC |
| TemptrsAR-Rv | CTTCGGTTGTATTGTATTTTTTCT |
| Temptra20-Fw | CTTTCGCATCATCTGTAAATC |
| Temptra20-Rv | CCAATTACCGAATGCTTCC |
| TemptraL-Fw | GTTCAAATTGCCGAAAGTATG |
| TemptraL-Rv | GTCATGCCACAAACTTTTGC |
| TemptraA_a_-Fw | GATGGAGATGTTAGAAGAAATG |
| TemptraA_a_-Rv | GAATTCCCTAGTTTTTGTTCAG |
|  |  |
| IRD labeled primer extension primers |  |
| Primext_UtrsA | /5IRD700/ TTAGCTTCAAAATGATAAATAGTC |
| Primext_UtrsR | /5IRD700/ CAAGCTAAACCACTTATCTTTC |
| Primext_Utrs22 | /5IRD700/ GCACTCCGCTTATTATTTTTTTC |
| Primext_Utrs24 | /5IRD700/ TTTCTGCTAAAATAACAATGTGC |
| Primext_UtrsAR | /5IRD700/ CGTCTATAGTATACAAGAATTTTTT |
| Primext_Utra20 | /5IRD700/ ACTACCCTCTAAAAATGTATACCAA |
| Primext_UtraL | /5IRD700/ GTCCTCCTAAAACTTTAAATAAGTC |
| Primext_UtraA_a_ | /5IRD700/ CGAAGAACCTTTTTAAAATATTTC |
|  |  |
| EMSA primers |  |
| Utra20-Fw | /5IRD700/ TCAACTAGCAATTCGGGTATAT |
| Utra20-Rv | /5IRD700/ TTAACCAAATTCGCCCATGTTT |
| Utra20-R1 | ACTACCCTCTAAAAATGTATACC |
| Utra20-R2 | CATTAATATTGCGTTTTGTTTACG |
| Utra20-R3 | TTGCAAGCGGACTGCTTC |
| Utra20-F4 | TTTGTTCATGCTCGTTGGTC |
| Utra20-F5 | AAAGGAATATAAACGTAAACAAAAC |
| Utra20-F6 | AGTTATAGAAAGGAGTAAACATTG |
| UtraL-Fw | /5IRD700/ AGAAGACATATCAGAAGCAAAG |
| UtraL-Rv | /5IRD700/ CCTACTTTAACAGTTGAACTAG |
| UtraL-R1 | GTCCATAAAATGAACCTCCTAA |
| UtraL-R2 | CGGTGCTAATAGTGATTTAAAC |
| UtraL-R3 | AAGCAAAGCTAATAATACTAGTC |
| UtraL-F4 | ACGGAGCGGTTGTAAGAG |
| UtraL-F5 | TTAGCTTTGCTTATTGGTGTTG |
| UtraL-F6 | CTATTAGCACCGATTTTAGTAAG |
| UtraL-F7 | TTAGGAGGTTCATTTTATGGAC |
| UtraA_a_-Fw | /5IRD700/ AGAAGAAATGATACAGGAACAATT |
| UtraA_a_-Rv | /5IRD700/ GATACCTGTTTCTTTCATCATC |
| UtraA_a_-R1 | TCTTGTCAACCTCTCAAATTTC |
| UtraA_a_-R2 | TCATCTTTCACTCACAAAAGC |
| UtraA_a_-R3 | TGGTGTCCCCCAATCCC |
| UtraA_a_-R4 | AACTTCCTCTTCGGTCAAAG |
| UtraA_a_-F5 | CCGAAGAGGAAGTTAAAAATTTG |
| UtraA_a_-F6 | GGATTGGGGGACACCAC |
| UtraA_a_-F7 | ACTAAGCTCGCTAAAGACAC |
| UtraA_a_-F8 | AATTTGAGAGGTTGACAAGATG |
| UtrsA-Fw | /5IRD700/CTTCTCCAATACGAGTATTAATG |
| UtrsA-Rv | /5IRD700/GCGTTCACTGTATAATTTTTCC |
| UtrsA-R1 | GAAGTGTTCTCCTTTCTTTTTTA |
| UtrsA-R2 | GTGCGCCCTTATATCTTATAG |
| UtrsA-R3 | GCCTTATTTTGTTGCATTTGATA |
| UtrsA-F4 | CTTTTTTTCTTTGCCATAACTTC |
| UtrsA-F5 | GCGCACTTATATACCATGAAA |
| UtrsA-F6 | CGCTTTGCTCGTGTAAAAAAT |
| UtrsA-F7 | AAAGTTATTTCAAGAGGGAAAG |
| UtrsR-Fw | /5IRD700/AAAATCCAAGCATGAAAGTTATG |
| UtrsR-Rv | /5IRD700/GAAAGTATTCTGGATGTCTTG |
| UtrsR-R1 | CCACTTATCTTTCGACATTTC |
| UtrsR-R2 | CATTTTTGGACTGTATCCTCTA |
| UtrsR-R3 | CCTTTGAGCAGATTGAAATTATT |
| UtrsR-R4 | CAGCTTTTAATGTTTGCTTGC |
| UtrsR-F5 | GTCAAAGATGAACTTAACAAAGA |
| UtrsR-F6 | GACTAGGAACAAATCTACTCTT |
| UtrsR-F7 | CCTGTCCTCATTTGTTGTATT |
| UtrsR-F8 | GTCGAAAGATAAGTGGTTTAG |
| Utrs22-Fw | /5IRD700/GAACAGTTAGAACGCTATAAAG |
| Utrs22-Rv | /5IRD700/CTTTTAAATTCCACTCTCCATTG |
| Utrs24-Fw | /5IRD700/GTGTCTTTCACGCTGAATG |
| Utrs24-Rv | /5IRD700/CGTTTGGGAAAAAATCATTGTC |
| UtrsAR-Fw | /5IRD700/GCCAAACGTTGACTATTTTCT |
| UtrsAR-Rv | /5IRD700/CGGTTGTCTTTTTGGATAATG |
|  |  |
| Conjugation screening primers |  |
| ScrnpNP40-Fw | TGTGGGAGAAGCTACTGGAGAATGGATACC |
| ScrnpNP40-Rv | TTGCTCGGACTGCCGGAATTGGTG |
| ScrnMG1614-Fw | AATGGCTCGGAGATGAAGAC |
| ScrnMG1614-Rv | ACAATTCCACGAAGGCTCTC |
| ScrnpUC11B-Fw | CGGTATCTTAGCACATGTTG |
| ScrnpUC11B-Rv | CATGGTTCAATTGTTGTTTGTAA |

* Phosphorothioate linkages of recombineering oligos.

ᴪ Introduced restriction enzyme sites are single underlined.

₳ Hexa His Tag sequences required for Ni-NTA purification are double underlined
