# Supplementary material for: Transcriptional control of two distinct lactococcal plasmid-encoded conjugation systems
Source: Curr Res Microb Sci. 2024 Feb 5;6:100224. doi: 10.1016/j.crmicr.2024.100224 (PMC10873654; doi:10.1016/j.crmicr.2024.100224)
Supplement: Supplementary file 4 [file mmc4.docx]

**Supplementary Table S4.** Locations and sizes of the different IRD700-labelled DNA fragments used in the Electrophoretic Mobility Shift assays, in relation to the promoter regions’ respective transcription starting sites. A positive result for a band shift is indicated by a ‘+’, while no observed shift is represented by a ‘-‘. Fragment positions are given in relation to the deduced transcription start site.

| **Fragment name** | **Fragment Start position** | **Fragment End position** | **Fragment size (bp)** | **Shift observed (+/-)** |
| --- | --- | --- | --- | --- |
| **Utra20** | -302 | +158 | 461 | + |
| **Utra20 R1** | -302 | +55 | 358 | + |
| **Utra20 R2** | -302 | -37 | 266 | - |
| **Utra20 R3** | -302 | -142 | 161 | - |
| **Utra20 F4** | -191 | +158 | 350 | + |
| **Utra20 F5** | -73 | +158 | 232 | + |
| **Utra20 F6** | -4 | +158 | 163 | - |
| **UtraL** | -401 | +124 | 526 | + |
| **UtraL R1** | -401 | +29 | 431 | + |
| **UtraL R2** | -401 | -68 | 334 | - |
| **UtraL R3** | -401 | -179 | 223 | - |
| **UtraL F4** | -276 | +124 | 401 | + |
| **UtraL F5** | -190 | +124 | 315 | + |
| **UtraL F6** | -79 | +124 | 204 | + |
| **UtraL F7** | +8 | +124 | 117 | - |
| **UtraA_a_** | -373 | +132 | 506 | + |
| **UtraA_a_ R1** | -373 | +31 | 405 | + |
| **UtraA_a_ R2** | -373 | -73 | 301 | - |
| **UtraA_a_ R3** | -373 | -147 | 227 | - |
| **UtraA_a_ R4** | -373 | -271 | 103 | - |
| **UtraA_a_ F5** | -284 | +132 | 417 | + |
| **UtraA_a_ F6** | -162 | +132 | 295 | + |
| **UtraA_a_ F7** | -42 | +132 | 175 | + |
| **UtraA_a_ F8** | +12 | +132 | 121 | - |
| **UtrsA** | -350 | +127 | 478 | + |
| **UtrsA R1** | -350 | +20 | 371 | + |
| **UtrsA R2** | -350 | -88 | 263 | - |
| **UtrsA R3** | -350 | -156 | 195 | - |
| **UtrsA F4** | -243 | +127 | 371 | + |
| **UtrsA F5** | -93 | +127 | 221 | + |
| **UtrsA F6** | -22 | +127 | 150 | - |
| **UtrsA F7** | +47 | +127 | 81 | - |
| **UtrsR** | -369 | +120 | 490 | + |
| **UtrsR R1** | -369 | +35 | 405 | + |
| **UtrsR R2** | -369 | -59 | 311 | - |
| **UtrsR R3** | -369 | -174 | 196 | - |
| **UtrsR R4** | -369 | -283 | 87 | - |
| **UtrsR F5** | -259 | +120 | 380 | + |
| **UtrsR F6** | -174 | +120 | 295 | + |
| **UtrsR F7** | -37 | +120 | 158 | + |
| **UtrsR F8** | +20 | +120 | 101 | - |
