# Supplementary material for: Transcriptional control of two distinct lactococcal plasmid-encoded conjugation systems
Source: Curr Res Microb Sci. 2024 Feb 5;6:100224. doi: 10.1016/j.crmicr.2024.100224 (PMC10873654; doi:10.1016/j.crmicr.2024.100224)
Supplement: Supplementary file 5 [file mmc5.docx]

**Supplementary Figure S1.** EMSA gel images of purified Tra20/TraR/TraA_b_ and TrsA/TrsR in increasing concentrations (0, 10, 50, 100, 150 and 250 nM), incubated with 0.1 pmol of IRD700-labelled PCR fragments containing P_20_, P_L_, P_Aa_, P_A_ or P_R_.


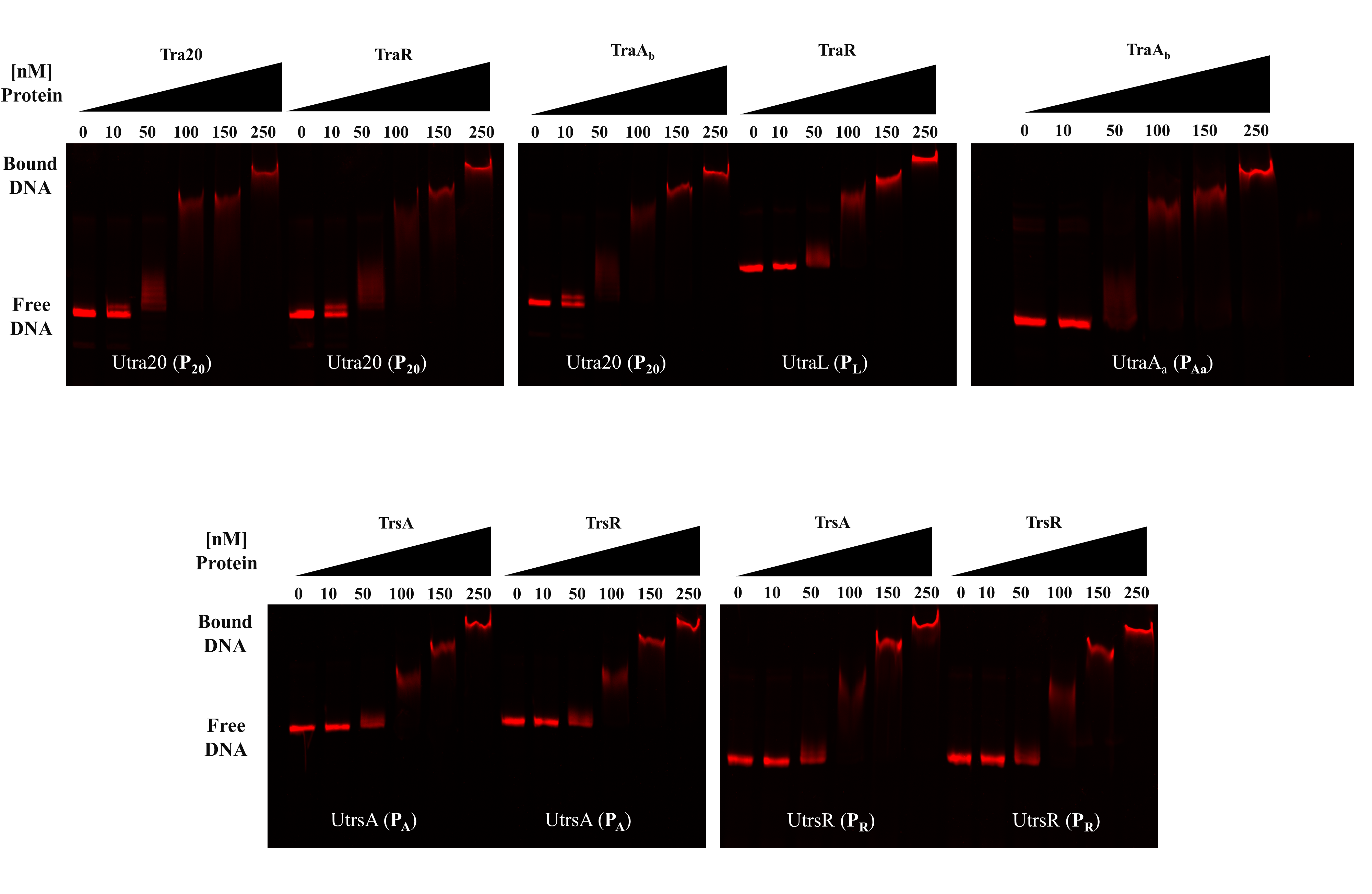


**Supplementary Figure S2.** EMSA gel images of purified Tra20/TraR/TraAb and TrsA/TrsR (250 nM), incubated with 0.1 pmol of differently sized IRD700-labelled PCR fragments located close to P_20_, P_L_ or P_Aa_ in pNP40, or close to P_A_ or P_R_ in pUC11B.
